# Connectivity Patterns of Deep Brain Stimulation Targets in Patients with Gilles de la Tourette Syndrome

**DOI:** 10.3390/brainsci11010087

**Published:** 2021-01-11

**Authors:** Petra Heiden, Mauritius Hoevels, Dilruba Bayram, Juan C. Baldermann, Thomas Schüller, Daniel Huys, Veerle Visser-Vandewalle, Pablo Andrade

**Affiliations:** 1 Department of Stereotactic and Functional Neurosurgery, University Hospital of Cologne, 50937 Cologne, Germany; mauritius.hoevels@uk-koeln.de (M.H.); veerle.visser-vandewalle@uk-koeln.de (V.V.-V.); pablo.andrade-montemayor@uk-koeln.de (P.A.); 2 Department of Neurosurgery, University Hospital of Cologne, 50937 Cologne, Germany; dilruba.bayram@web.de; 3 Department of Psychiatry and Psychotherapy, University Hospital of Cologne, 50937 Cologne, Germany; juan.baldermann@uk-koeln.de (J.C.B.); thomas.schueller@uk-koeln.de (T.S.); daniel.huys@uk-koeln.de (D.H.); 4 Department of Neurology, University Hospital of Cologne, 50937 Cologne, Germany

**Keywords:** Tourette syndrome, deep brain stimulation, tractography, normative connectome, globus pallidus internus, thalamus

## Abstract

Since 1999, several targets for deep brain stimulation (DBS) in Gilles de la Tourette syndrome (GTS) have emerged showing similar success rates. Studies using different tractography techniques have identified connectivity profiles associated with a better outcome for individual targets. However, GTS patients might need individualized therapy. The objective of this study is to analyze the connectivity profile of different DBS targets for GTS. We identified standard target coordinates for the centromedian nucleus/nucleus ventro-oralis internus (CM/Voi), the CM/parafascicular (CM-Pf) complex, the anteromedial globus pallidus internus (amGPi), the posteroventral GPi (pvGPi), the ventral anterior/ventrolateral thalamus (VA/VL), and the nucleus accumbens/anterior limb of the internal capsule (Nacc/ALIC). Probabilistic tractography was performed from the targets to different limbic and motor areas based on patient-specific imaging and a normative connectome (HCP). Our analysis showed significant differences between the connectivity profiles of standard DBS targets (*p* < 0.05). Among all targets, the pvGPi showed the strongest connection to the sensorimotor cortex, while the amGPi showed the strongest connection to the prefrontal cortex in patient-specific imaging. Differences were observed between the connectivity profiles when using probabilistic tractography based on patient data and HCP. Our findings showed that the connectivity profiles of different DBS targets to major motor and limbic areas differ significantly. In the future, these differences may be considered when planning DBS for GTS patients employing an individualized approach. There were compelling differences in connectivity profiles when using different tractography techniques.

## 1. Introduction

Gilles de la Tourette syndrome (GTS) is a chronic neuropsychiatric disorder that typically presents with a combination of vocal and motor tics [1]. First symptoms usually surface in early childhood, reaching peak intensity around the age of 10, then gradually diminishing during late adolescence and early adulthood [2,3]. About 20–30% of GTS patients do not experience a spontaneous improvement and can remain severely affected [4]. In some patients, the severity and the frequency of tics increases with time. GTS is frequently associated with psychiatric comorbidities, such as attention deficit disorder, obsessive-compulsive disorder, and self-injurious behavior, among others [5].

The pathophysiology behind GTS is still not fully understood. Studies researching functional and anatomical connectivity in GTS patients have shown immature patterns in the cortico-basal ganglia-thalamo-cortical (CBTC) loops, with stronger, more disorganized connections between regions in close proximity and weaker connectivity between distant cerebral areas [6,7,8].

Treatment for GTS consists of cognitive behavioral therapy and pharmacotherapy, which usually contribute to a significant reduction in symptoms [9]. For patients with treatment refractory and disabling tics, deep brain stimulation (DBS) can be considered a therapeutic option. In 1999, the first report describing DBS for GTS was published by Vandewalle et al. [10]. In this study and their subsequent reports, they targeted the centromedian nucleus (CM), the substantia periventricularis (Svp), and the nucleus ventro-oralis internus (Voi) in the thalamus, successfully reducing the frequency and severity of tics [11]. Since then, several structures along the CBTC loops have been researched as DBS targets for GTS: the CM-parafascicular (Pf) complex [12], the anteromedial globus pallidus internus (amGPi) [12], the posteroventral globus pallidus internus (pvGPi) [13], the ventral anterior/ventrolateral thalamus (VA/VL) [14], and the nucleus accumbens/anterior limb of the internal capsule (Nacc/ALIC) [15]. Individual case studies have reported clinically effective stimulation in the globus pallidus externus (GPe) [16] and the subthalamic nucleus [17]. Several reports studying the clinical outcome of GTS patients treated with DBS have shown a significant improvement in tics with all above-mentioned targets, achieving on average a comparable degree of improvement [18,19]. However, there is a great discrepancy between the response of individual patients in all cohorts, with some of them showing almost complete reduction in tics and some barely responding to the therapy [18]. 

Recent studies have reported a correlation between the connectivity patterns of different DBS targets and a reduction in the symptoms of GTS patients [20,21,22]. However, most studies have focused on a single DBS target, with only one study comparing pallidal and thalamic stimulation [22]. The analyses were based either on normative structural connectome atlases or on patient-specific diffusion-weighted MRI, which complicates the comparison of the findings. In summary, an overview of the connectivity patterns of different DBS targets in GTS is still lacking.

The aim of this study was to analyze the connectivity profiles of all the standard GTS DBS targets available and compare the connectivity profiles between data based on patient-specific diffusion-weighted MRI of GTS patients and a normative connectome. In addition, we compared the differences in the connectivity profiles based on clinical response.

## 2. Materials and Methods

### 2.1. Patient Data and Study Design

We retrospectively analyzed the data of seven patients with GTS who underwent surgery for thalamic DBS at the University Hospital of Cologne between 2016 and 2018. The patients were diagnosed according to the criteria of the fifth edition of the Diagnostic and Statistical Manual of Mental Disorders (DSM-V). All patients received diffusion tensor imaging as part of preoperative MRI. In all patients, DBS electrodes were implanted in the CM/Voi bilaterally, according to standard coordinates [11], and adapted to the patients’ individual anatomy. Clinical outcome was assessed using the Yale Global Tic Severity Scale (YGTSS) [23] prior to surgery and six months after the procedure. 

### 2.2. Volume of Tissue Activated and Seed Regions

The coordinates, relative to the midpoint of the line connecting the anterior with the posterior commissure (AC-PC), for standard targets of DBS in GTS were determined according to scholarly literature:

- CM/Voi: *x* = +/− 5 mm, *y* = −4 mm, *z* = 0 mm [11,24]
- CM-Pf: *x* = +/− 6 mm, *y* = −10.2 mm, *z* = 1.2 mm [12,25]
- VA/VL: *x* = +/− 7.1 mm, *y* = −5.7 mm, *z* = −1.8 mm [14]
- amGPi: *x* = +/− 12 mm, *y* = 7.5 mm, *z* = −3.0 mm [12,25,26]
- pvGPi: *x* = +/− 20 mm, *y* = 3.5 mm, *z* = −4.0 mm [13,24,27]
- Nacc/ALIC: *x* = +/− 6.5 mm, *y* = 15.2 mm, *z* = 4.5 mm [15,28]

**Figure 1:**
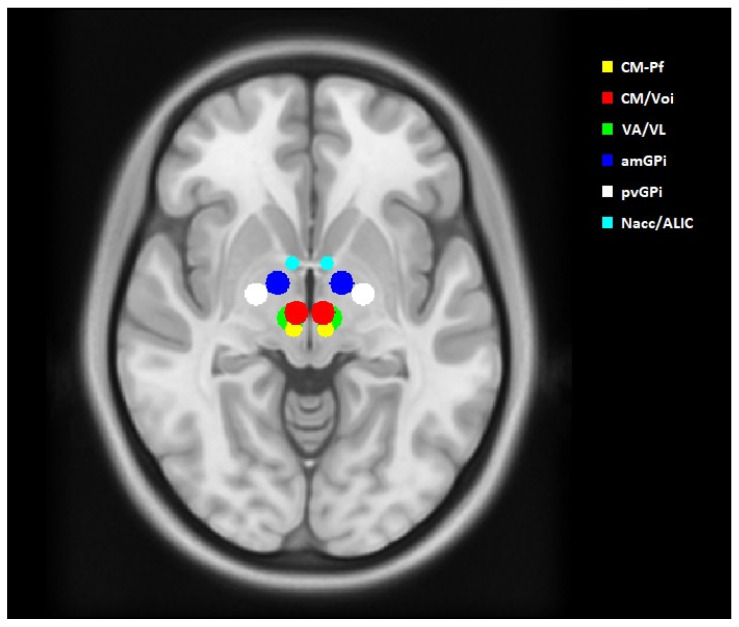
Anatomical location of the sham volumes of activated tissue (VTAs) in the ICBM 152 MNI 2009b space in axial view. All VTAs are spherical, with a diameter of 5 mm.

The coordinates were transferred to the standard asymmetric stereotactic space, using ICBM 152 MNI 2009b (MNI) [29]. We created sham volumes of tissue activated (VTAs) with a diameter of 5 mm in the MNI space at these targets. The sizes of the sham VTAs were derived from the actual stimulation parameters and postoperative CT scans of three of our GTS patients with the best clinical outcome (Table 1). Using the open-source software Lead-DBS (www.lead-dbs.org, Charité University Hospital, Berlin, Germany), the VTAs were estimated for these patients. The procedure has been described in detail in our previous publication [21]. The sham VTAs were transferred to each patient’s T2 sequence using FLIRT from the FSL v6.0 program (FMRIB Software Library, www.fmrib.ox.ac.uk/fsl) for affine linear matching, followed by deformable registration by advanced normalization tools (ANTs, http://stnava.github.io/ANTs/) [30]. The generated VTAs are shown in Figure 1. 

Based on scholarly literature, we defined the prefrontal cortex, the primary motor cortex (M1), the primary sensory cortex (S1), the supplementary motor area (SMA), the pre-SMA, the amygdala, and the hippocampus as our seed regions [7,21,31]. M1, S1, and the prefrontal cortex were defined manually in the MNI space. For the identification of SMA and pre-SMA, we used the human motor area template [32]. The amygdala and the hippocampus were determined based on the MNI PD25 atlas [33]. We transferred the seed regions to the patients’ T2 sequence using the same procedure as described above. Subsequently, the seed regions were manually adapted to the individual anatomy of the patients based on T1 and T2 sequences using the editing tools of the FSLeyes image viewer. 

### 2.3. Probabilistic Tractography

The DTIs were corrected for EPI distortions, subject movement, and eddy current distortions using the TOPUP and EDDY tools from FSL [34,35,36,37]. Probabilistic fiber tracking was performed from the VTAs to the ipsilateral seed regions using the Oxford Centre for Functional MRI of the Brain (FMRIB) FSL probtrackx2 program [38,39]. We applied the seed regions as binary masks onto the respective tracts using the fslmaths tool and quantified the mean intensity of the voxels localized within the seed regions for the measurement of the tract density. As a further indicator of connectivity, the number of streamlines connecting the VTAs with the seed regions was calculated using the FSL tracking tool (probtrackx2) in network mode. 

Additionally, the connectivity of the VTAs to the seed regions was analyzed in a structural group connectome based on the diffusion spectrum imaging of 32 healthy adult subjects (HCP) [40,41] using TrackVis imaging software. 

### 2.4. Statistical Analysis 

All data were analyzed using SPSS (IBM Corp. Released 2020. IBM SPSS Statistics for Macintosh, Version 27.0. Armonk, New York, NY, USA). Each brain hemisphere was analyzed separately, creating two separate datapoints per fiber tract per patient. As normal distribution could not be assumed (Kolmogorov-Smirnov test), the Kruskal-Wallis test was conducted to compare the connectivity parameters of each VTA with the individual seed regions. Post hoc analysis was conducted using the Dunn-Bonferroni test. Responders and non-responders were compared using the Mann-Whitney test. *p*-values under 0.05 were considered significant. The data supporting the findings of this study, such as the DBS MRI datasets, are not publicly available due to data privacy regulations, but are available from the corresponding author upon reasonable request.

## 3. Results

### 3.1. Patient Data

The mean age at the date of the surgery was 30 years (±9.1 years), ranging from 20 to 46 years. All patients but one were male. All patients had severe GTS preoperatively with a mean YGTSS of 90 (±8.8). Four out of seven patients presented with psychiatric comorbidities, the most common being depression and obsessive-compulsive disorder (OCD) (*n* = 2 for both). There was a significant clinical improvement six months after the surgery, with a mean YGTSS score of 52 (±21.5) (*t* = 6.921, *p* < 0.001). A minimum of 35% improvement in YGTSS was defined as a clinical response. Five patients were considered responders and two as non-responders.

### 3.2. Connectivity Analysis 

#### 3.2.1. Image Processing Based on Patient-Specific Data 

##### Streamline Count

**Figure 2:**
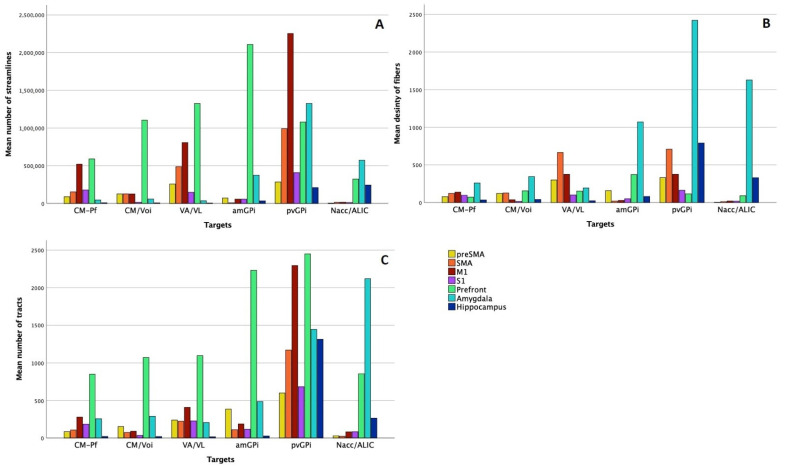
Comparison of connectivity patterns of standard deep brain stimulation (DBS) targets in Gilles de Tourette syndrome (GTS) using different techniques. Diagram **A** and Diagram **B** show our analysis using patient-specific imaging. Diagram A summarizes our findings regarding the number of streamlines projecting from the DBS targets to the selected cortical and subcortical areas. Diagram B displays the mean density of fibers in the seed regions of tracts projecting from different targets. Diagram **C** shows the connectivity profiles of the standard targets in a normative connectome based on healthy subjects.

There was a significant difference between the number of streamlines connecting each VTA to individual seed regions (pre-SMA: chi-square = 36.53, *p* < 0.001; SMA: chi-square = 53.42, *p* < 0.001; M1: chi-square = 55.12, *p* < 0.001; S1: chi-square = 41.38, *p* < 0.001; prefrontal cortex: chi-square = 16.77, *p* = 0.005; amygdala: chi-square = 52.11, *p* < 0.001; hippocampus: chi-square = 37.1, *p* < 0.001; *df* = 5) (Figure 2A). The highest number of tracts connecting to the sensorimotor cortex projected from the pvGPi (mean number of streamlines: M1: 2,252,929.04 ± 1,740,254.85; SMA: 995,187.57 ± 762,682.91; pre-SMA: 284,831.46 ± 380,020.22; S1: 407,883.68 ± 271,275.25). The amGPi had the most streamlines connecting to the prefrontal cortex (mean number of streamlines: 2,107,467.07 ± 259,811.28). The Nacc/ALIC showed the strongest connection to the hippocampus (mean number of streamlines: 243,571.79 ± 378,577.93), and the pvGPi to the amygdala (mean number of streamlines: 1,325,948.14 ± 431,272.06). Comparing the thalamic targets, the VA/VL had the most tracts connecting to the motor cortex (mean number of streamlines: M1: 807,011.14 ± 762,080.53; SMA: 487,448.86 ± 398,613.72; pre-SMA: 257,989.57 ± 345,232.07) and to the prefrontal cortex (mean number of streamlines: 1,325,091.14 ± 1,304,141.08). The pvGPi had significantly more streamlines connecting to M1 and SMA in comparison to the amGPi (*p* < 0.001).

**Figure 3:**
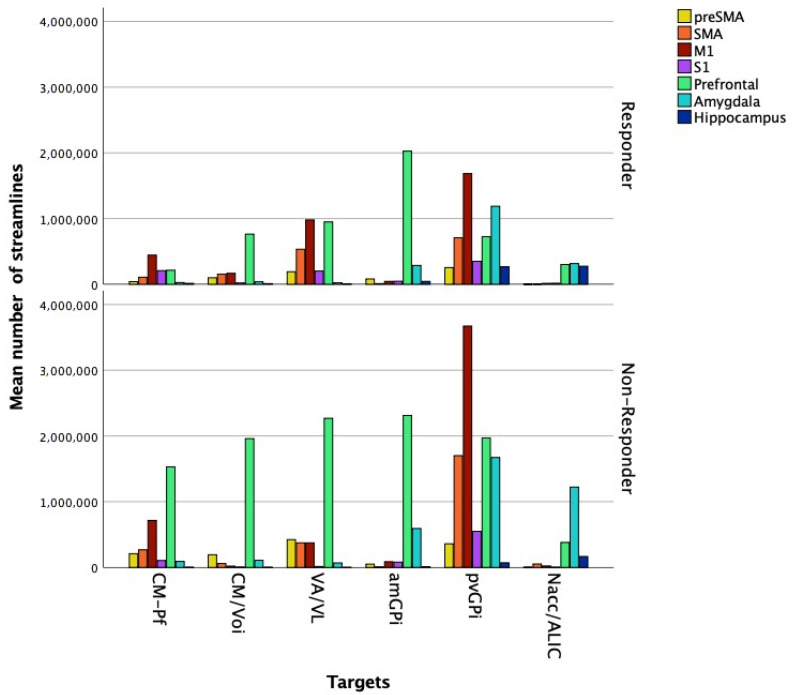
Diagram summarizing the mean number of streamlines from each target to the different seed regions in responder and non-responder patients. Datapoints represent segmented analysis of the hemispheres (responders, *n* = 10; non-responders, *n* = 4).

Comparing responders to non-responders, there was a significant difference between the mean number of streamlines from Nacc/ALIC to amygdala (responders: 314,632.65 ± 193,876.50; non-responders: 1,221,076.75 ± 414,805.22, *U* = 0, *p* = 0.02) (Figure 3).

##### Fiber Density

To measure the degree of connectivity, we compared the mean density of the fiber tracts projecting from each VTA within the seed regions. This analysis showed significant differences between the VTAs (pre-SMA: chi-square = 38.95, *p* < 0.001; SMA: chi-square = 57.82, *p* < 0.001; M1: chi-square = 53.01, *p* < 0.001; S1: chi-square = 34.85, *p* < 0.001; prefrontal cortex: chi-square = 21.63, *p* < 0.001; amygdala: chi-square = 50.45, *p* < 0.001; hippocampus: chi-square = 25.82, *p* < 0.001; *df* = 5) (Figure 2B). The mean density of the fibers projecting to the motor cortex was the highest in the pvGPi (M1: 376.18 ± 190.78; SMA: 707.38 ± 632.63; pre-SMA: 331.38 ± 433.51) and almost identical in the VA/VL (M1: 374.43 ± 299.08; SMA: 664.15 ± 549.51; pre-SMA: 298.29 ± 286.01). The fibers projecting from the amGPi had the highest density in the prefrontal cortex (mean tract density: 371.67 ± 274.80). The pvGPi showed the strongest connection to the hippocampus (mean tract density: 790.31 ± 1878.79) and to the amygdala (mean tract density: 2421.24 ± 907.29). 

**Figure 4:**
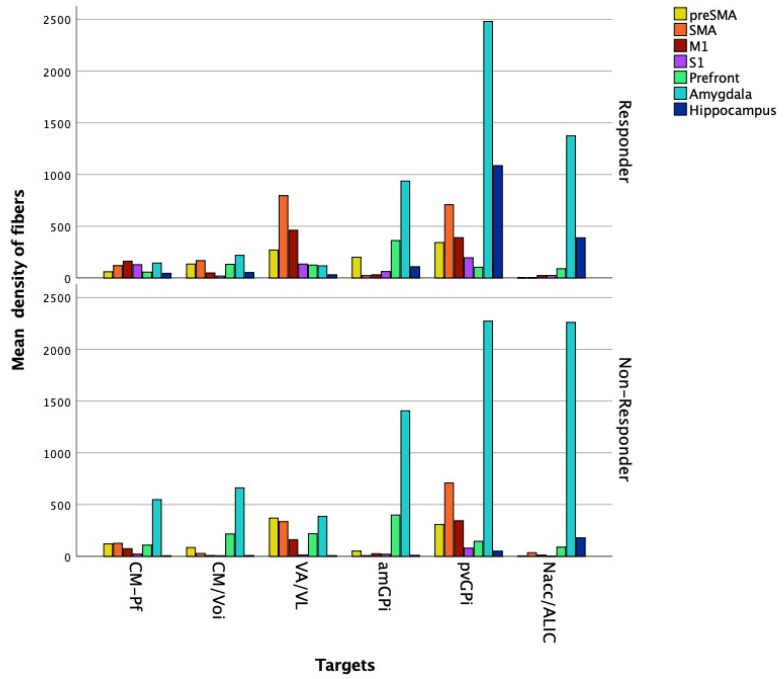
Diagram summarizing the mean density of fibers in the seed regions of the tracts projecting from the individual targets in responder and non-responder patients. Datapoints represent segmented analysis of the hemispheres (responders, *n* = 10; non-responders, *n* = 4).

There was a significant difference between responders and non-responders in the mean density of the fibers in S1 connecting to the CM-Pf (responders: 127.73 ± 94.62; non-responders: 21.9 ± 14.58; *U* = 5, *p* = 0.036) (Figure 4).

#### 3.2.2. Image Processing Based on a Normative Connectome 

To examine the connectivity pattern of the standard DBS targets in healthy subjects, we analyzed the number of tracts connecting the VTAs to the seed regions in HCP. There was no significant difference between the number of tracts projecting to the seed regions’ VTAs (pre-SMA: chi-square = 9.62, *p* = 0.09; SMA: chi-square = 8.31, *p* = 0.14; M1: chi-square = 8.39, *p* < 0.14; S1: chi-square = 5.89, *p* = 0.32; prefrontal cortex: chi-square = 8.31, *p* = 0.14; amygdala: chi-square = 9, *p* = 0.11; hippocampus: chi-square = 8.63, *p* = 0.13; *df* = 5) (Figure 2C). Similar to the connectivity patterns in patient data, the pvGPi had the strongest connectivity to the sensorimotor cortex (mean number of tracts: M1: 2294.5 ± 300.52; SMA: 1169 ± 219.20; pre-SMA: 599 ± 251.73; S1: 683.5 ± 149.20). The pvGPi also showed the strongest connectivity to the prefrontal cortex (mean number of tracts: 2450 ± 1217.63) and the hippocampus (mean number of tracts: 1312 ± 1275.62). The most tracts to the amygdala projected from the Nacc/ALIC (mean number of tracts: 2119.5 ± 127.28). Within the thalamic targets, the VA/VL showed the strongest connectivity to the motor cortex (mean number of tracts: M1: 408.5 ± 354.26; SMA: 223 ± 120.21; pre-SMA: 239.5 ± 37.48). 

## 4. Discussion

Our study shows significant differences between the connectivity patterns of standard DBS targets for GTS. According to our results, the pvGPi showed the highest connectivity to the sensorimotor cortex from all DBS targets, both in the analysis based on patient-specific data and in HCP. In regard to the thalamic targets, the VA/VL showed particularly high connectivity to the motor cortex both in patient-based analysis and in the normative connectome. With respect to the prefrontal cortex, the amGPi showed the highest connectivity in patient-specific data, while the pvGPi showed a higher connectivity in HCP. Most fibers that connect to the amygdala projected from the pvGPi when analyzing patient-specific imaging. However, in HCP, the amygdala had the strongest connectivity to the Nacc/ALIC. Within the thalamic targets, the CM/Voi showed the strongest connection to the amygdala both in patient-specific data and in HCP. The insights from these analyses may prove helpful when choosing the ideal DBS target for individual patients. For instance, previous studies showed abnormal activity in the amygdala and the hippocampus in OCD patients [42,43]. Therefore, based on these findings, when choosing between thalamic targets, the CM/Voi might prove more beneficial for GTS patients with strong OCD components. Otherwise, based on previous functional imaging studies [44], the VA/VL might be preferred for patients with severe tics and without psychiatric comorbidities, based on its connectivity to the motor cortex and the prefrontal cortex. For a specific stimulation of tracts connecting to the prefrontal cortex, the amGPi was shown to be more suitable, while the pvGPi is more eligible for diffuse stimulation of tracts connecting to several cortical and subcortical regions.

**Figure 5:**
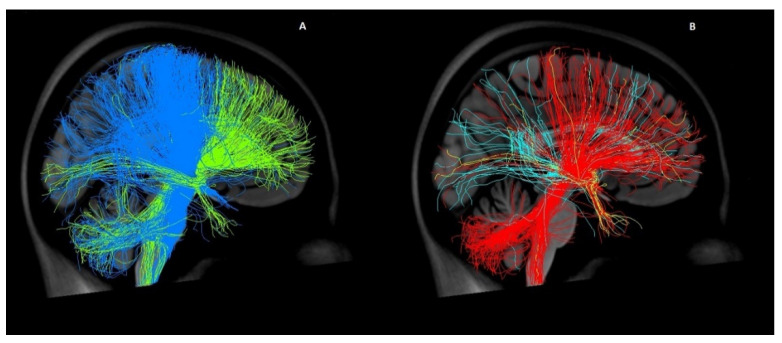
Comparison of the connectivity profiles of different DBS targets in GTS. Both figures show fiber tracts from the selected targets connecting to the entire brain in a three-dimensional lateral view from the left in the MNI space using a normative connectome. (**A**) displays tracts from the posteroventral globus pallidus internus (pvGPi) in blue and tracts from the anteromedial globus pallidus internus (amGPI) in green. In (**B**), the tracts from the ventral anterior/ventrolateral thalamus (VA/VL) are represented in red, the tracts from the centromedian nucleus/parafascicular (CM-Pf) in light blue, and the tracts from the CM/ventro-oralis internus (CM/Voi) in yellow.

Our analysis showed significant differences within the pallidal and also within the thalamic targets (Figure 5). In previous tractography studies, the CM-Pf and the CM/Voi were merged into one target, and in some cases the two pallidal targets were also regarded as one [19,22]. Based on our findings, all targets must be analyzed separately to provide a recommendation for lead placement or targeted stimulation.

The results of our study also showed differences between the connectivity patterns of the DBS targets in patient-specific imaging versus HCP. For instance, in the GPi, patient-specific data showed a stronger connectivity from the amGPi to the prefrontal cortex than from the pvGPi. However, in HCP, the ratio was the opposite. The connectivity patterns of the targets in patient-specific imaging showed significant differences; however, the differences in HCP were not significant. Because of technical and methodological differences, a direct comparison is not possible. For this purpose, larger patient groups and comparable DTI protocols would be necessary for better contrast. Normative connectomes are easily accessible and enable widespread usage, while diffusion imaging is more costly and especially challenging to acquire for patients with motor disorders. Previous research comparing normative connectomes to patient-specific imaging in patients with Parkinson’s disease and obsessive-compulsive disorder has proved that both techniques are valuable when analyzing connectivity profiles [45,46,47]. However, several studies showed impaired structural and functional connectivity in GTS [6,7,8], which should be considered when analyzing connectivity patterns in this patient group. 

Differences between responders and non-responders were observed. However, there were only a few tracts that showed significant discrepancies. Keeping the small number of participants in mind, these discrepancies could indicate a difference in structural connectivity in these two patient groups. Further studies with larger patient groups are necessary to evaluate whether certain connectivity patterns might be associated with better clinical outcome, or whether certain abnormalities in structural connectivity respond better to certain targets.

## 5. Conclusions

In our study, we report significant differences between the connectivity patterns of diverse standard DBS targets in GTS. These differences can assist in choosing targets for individual GTS patients based on different clinical patterns and/or comorbidities. We observed differences when comparing connectivity patterns using patient-specific data and a normative connectome. In addition, we observed differences in connectivity profiles between responders and non-responders, suggesting a distinct structural connectivity between these groups.

## Figures and Tables

**Table 1:** Summary of the stimulation parameters of the three patients with the best clinical outcome.

Patient Number	Frequency (Hz)	Pulse Width (µs)	Amplitude (V)
Patient 1	130	90	3.2
Patient 2	100	90	3.0
Patient 3	130	90	3.3

## Data Availability

The data presented in this study are available on request from the corresponding author. The data are not publicly available due to data privacy regulations.
